# Postnatal expression of Cat-315-positive perineuronal nets in the SAMP10 mouse primary somatosensory cortex

**DOI:** 10.1016/j.ibneur.2025.01.012

**Published:** 2025-01-18

**Authors:** Hiroshi Ueno, Yu Takahashi, Sachiko Mori, Eriko Kitano, Shinji Murakami, Kenta Wani, Yosuke Matsumoto, Motoi Okamoto, Takeshi Ishihara

**Affiliations:** a Department of Medical Technology, Kawasaki University of Medical Welfare, Okayama 701-0193, Japan; b Department of Psychiatry, Kawasaki Medical School, Kurashiki 701-0192, Japan; c Department of Neuropsychiatry, Graduate School of Medicine, Dentistry and Pharmaceutical Sciences, Okayama University, Okayama 700-8558, Japan; d Department of Medical Technology, Graduate School of Health Sciences, Okayama University, Okayama 700-8558, Japan

**Keywords:** Ageing, Brain function, Neuroplasticity, Neuroprotection, Cognitive decline

## Abstract

Perineuronal nets (PNNs) form at the end of the critical period of plasticity in the mouse primary somatosensory cortex. PNNs are said to have functions that control neuroplasticity and provide neuroprotection. However, it is not clear which molecules in PNNs have these functions. We have previously reported that Cat-315-positive molecules were not expressed in the PNNs of the senescence-accelerated model (SAM)P10 strain model mice at 12 months of age. To confirm whether the loss of Cat-315-positive molecules occurred early in life in SAMP10 mice, we examined Cat-315-positive PNNs in the primary somatosensory cortex during postnatal development. This research helps to elucidate the function of PNNs and the mechanism of cognitive decline associated with ageing. To confirm whether Cat-315-positive PNNs changed in an age-dependent manner in SAMP10 mice, we examined the primary somatosensory cortex at 21, 28, and 56 days after birth. We compared these results with those of senescence-accelerated mouse-resistant (SAMR) mice. In SAMP10 mice, Cat-315-positive PNNs were expressed in the primary somatosensory cortex early after birth, but their expression was significantly lower than that in SAMR1 mice. Many other molecules that calibrated the PNN were unchanged between SAMP10 and SAMR1 mice. This study revealed that the expression of the Cat-315 epitope was decreased in the primary somatosensory cortex of SAMP10 mice during postnatal development. SAMP10 mice have had histological abnormalities in their brains since early life. Furthermore, using SAMP10 will be useful in elucidating the mechanism of age-related abnormalities in brain function as well as in elucidating the function and structure of PNNs.

## Highlights

• Senescence-accelerated model (SAM)P10 mice have early brain abnormalities.
• Cat-315 epitope expression reduced during postnatal development in SAMP10.
• SAMP10 may help elucidate mechanisms of age-related abnormalities in brain function.

## Introduction

Ageing is an inevitable biological process characterised by a gradual decline in physiological functions, which can lead to a variety of age-related disorders. A decline in cognitive function, including memory and learning ability, is a well-known age-related disease that, as it progresses, can lead to dementia. Senescence-accelerated mice (SAMs) are established models for studying human ageing and age-related disorders. The SAM is a group of related inbred strains with 10 strains of senescence-accelerated (SAMP) mice and five strains of senescence-accelerated resistant (SAMR) mice (Takeda et al., 1981). Each SAMP strain has a strain-specific, age-related pathological phenotype (Takeda, 1999). In particular, SAMP8 and SAMP10 show learning and memory impairments associated with aging. Of the 10 SAMP mouse strains, the SAMP10 mouse strain was established by Shimada et al. (Shimada et al., 1994). Age-dependent decline in learning performance and memory has also been reported in SAMP10 mice (Shimada et al., 1993). SAMP10 brains exhibit age-related morphological changes, including dendritic regression, decreased dendritic spine density, synapse loss, and learning and memory impairments (Shimada et al., 1993, Shimada et al., 2003, Shimada et al., 2006). Therefore, the SAMP10 strain is a useful model for age-dependent, progressive neurological dysfunction. SAMP10 mice develop brain atrophy with age, and atrophy has been reported in the cerebral cortex analyzed in this study (Shimada et al., 1994).

The extracellular matrix (ECM) plays an important role in various physiological processes of the central nervous system (CNS) (Soleman et al., 2013, Frischknecht and Gundelfinger, 2012). In the CNS of adult mammals, various types of chondroitin sulfate proteoglycans (CSPGs) that bind chondroitin sulfate chains as glycosaminoglycans are present in the extracellular matrix (Siebert et al., 2014). Proteoglycans, a family of proteins with sulfated glycosaminoglycans, are major complex carbohydrates on the cell surface and in the extracellular matrix. Proteoglycans combine with many other extracellular matrix components and growth factors through their core proteins and glycosaminoglycan moieties. They are currently thought to play important roles in the regulation of cell adhesion, growth, motility, and differentiation (Schwartz, Domowicz, 2004). In particular, perineural nets (PNNs), specialised ECM structures that surround neuronal cell bodies and proximal dendrites in the CNS, control neuronal plasticity (Wang et al., 2012). PNNs are composed of hyaluronic acid, chondroitin sulfate proteoglycans (CSPGs), linked proteins, and tenascin-Rs. PNNs are expressed around parvalbumin (PV)-positive gamma-aminobutyric acid (GABA)-ergic interneurons and some pyramidal neurons in the cortex (Alpár et al., 2006, Ohyama and Ojima, 1997, Morris and Henderson, 2000). PNN development occurs during a critical period of brain maturation and is controlled by synaptic activity within the network (Nowicka et al., 2009, Carulli et al., 2010). In layer IV of the somatosensory cortex, perineural networks are formed at the end of a critical period of plasticity (Köppe et al., 1997). During postnatal cerebral cortical development, the termination of the critical period, a period of high plasticity, coincides with the accumulation of PNNs enveloping fast-spiking parvalbumin (PV) interneurons (Hensch, 2005). A significant portion of neuroplastic processes consistently solidified by the end of the first postnatal month in the somatosensory cortex of the mice. (Fox, 2002).PNN reduction is accompanied by the reinitiation of plasticity, regardless of the paradigm used (Lensjø et al., 2017, Fagiolini et al., 2004). Conversely, PNN stability is associated with memory resilience, and PNN deficits are thought to contribute to circuit dysfunction in several CNS pathologies (Testa et al., 2019).

PNNs are highly heterogeneous in their molecular composition and glycan structure of CSPGs. In many studies, Wisteria floribunda agglutinin (WFA), a plant lectin that exhibits preferential reactivity with glycans containing terminal N-acetylgalactosamine (GalNAc) residues, is routinely used to visualise PNNs (Härtig et al., 1992). The monoclonal antibody Cat-315 reacts with aggrecan to label the PNN population. Cat-315 recognises the human natural killer-1 (HNK-1) glycan epitope on aggrecan (Yamada, Jinno, 2017). Although the overall structure of the Cat-315 epitope is unknown, it is believed that the Cat-315 epitope is an O-mannose linked-oligosaccharide chain (Matthews et al., 2002). O-mannosyl glycan is a rare oligosaccharide found in mammals. O-mannosyl glycan, which binds α-dystroglycan, is known to play an important role in the molecular interactions between α-dystroglycan and its binding partners at the neuromuscular junction (Endo, 2005). In a previous study, we showed that expression of the Cat-315 epitope was absent in a 12-month-old SAMP10 strain (Ueno et al., 2023). It has also been reported that the expression of the Cat-315 epitope is lower in the 3-month-old SAMP10 strain than in the SAMR1 strain (Saitoh et al., 2008). However, the expression status of the Cat-315 epitope early in life remains unclear. Elucidating the expression of the Cat-315 epitope on aggrecan may help elucidate the mechanism of neuroplasticity. Examining age-dependent changes in the brains of the SAMP10 lineage will help elucidate the causes of age-related neurodegenerative diseases.

In this study, we investigated PNN in the primary somatosensory cortex of SAMP10 mice during postnatal development to obtain information on the Cat-315 epitope, a component of PNN that is expressed in an age-dependent manner. Developmental plasticity is well established in the rodent somatosensory cortex (Fox, 2002), so we undertook the present experiments to gain further insight into the role of PNNs in the somatosensory cortex. Experience-dependent developmental plasticity is well established in the mouse sensory cortex (Gordon and Stryker, 1996, Espinosa and Stryker, 2012, McRae et al., 2007, Nowicka et al., 2009), we used this system to investigate the role of PNNs and the expression of specific PNN components in SAMP10 mice during postnatal development.

## Materials and methods

### Ethics statements

All animal experiments were performed in accordance with the ARRIVE guidelines (https://www.nc3rs.org.uk/arrive-guidelines) and the U.S. National Institutes of Health's (NIH) Guide for the Care and Use of Laboratory Animals (NIH Publication No. 80–23, revised in 1996). This study was approved by the Committee for Animal Experiments at Kawasaki Medical School Advanced Research Centre. All efforts were made to minimise the number of animals used and their suffering. The use of animals was reduced via the experimental design, allowing statistically significant changes to be demonstrated, with the smallest number of animals per group and the smallest number of groups, which is consistent with scientific rigour.

### Animals

21-day-old SAMR1 and SAMP10 male mice were purchased from Japan SLC (Shizuoka, Japan) and housed individually in cages with food and water provided ad libitum under a 12 h light/dark cycle at 23–26 °C. Mice were randomly divided into three groups, a P21, P28 and P56 group (n = 5). In this experiment, mice were used that were reared until 28 or 56 day-old. Transparent plastic cages (220 × 340 × 150 mm) with wire tops were used, and a nonwoven filter cap was attached to the top of the wire. The cages included the provision of nesting material with food (MF-R; ORIENTAL YEAST, Tokyo, Japan) and water ad libitum, under 12-h light/dark conditions (lights on at 8:00, lights off at 20:00), with a temperature maintained between 23°C and 26°C.

### Tissue preparation

The mice were anesthetised with a lethal dose of sodium pentobarbital (120 mg/kg, i.p.) and transcardially perfused with 25 mL of phosphate-buffered saline (PBS), followed by 100 mL of 4 % paraformaldehyde in PBS (pH 7.4). Brains were dissected and fixed overnight at 4 °C in a fixative, cryopreserved in 15 % sucrose for 12 h, and preserved in 30 % sucrose for 20 h at 4 °C. Next, the brains were frozen in an optimum cutting-temperature compound (Tissue-Tek; Sakura Finetek, Tokyo, Japan) using a slurry of normal hexane in dry ice. Serial coronal sections with a thickness of 40 µm were obtained at −20 °C using a cryostat (CM3050S; Leica Wetzlar, Germany). Finally, the sections were collected in ice-cold PBS containing 0.05 % sodium azide.

### Immunohistochemistry

The sections were treated with 0.1 % Triton X-100 and PBS at room temperature for 15 min. After three washes with PBS, sections were incubated with 10 % normal goat serum (ImmunoBioScience Corp., Mukilteo, WA, USA) in PBS at room temperature for 1 h. They were then washed three times with PBS and incubated overnight at 4 °C in PBS containing biotinylated WFA (B-1355, Vector Laboratories; 1:200) and the antibodies described in the subsequent text. Subsequently, the sections were incubated with Alexa Fluor 594-conjugated streptavidin (S11227; Molecular Probes, Eugene, OR, USA) and the corresponding secondary antibodies (described in the Lectins and Antibodies subsection) at room temperature for 2 h. The labelled sections were rinsed with PBS and mounted on glass slides using Vectashield medium (H-1400; Vector Laboratories, Funakoshi Co., Tokyo, Japan). The prepared slides were stored at 4 °C until further microscopic analysis.

### Lectins and antibodies

The following lectins and primary antibodies were used for staining: biotinylated WFA (B-1355, Vector Laboratories; 1:200), mouse anti-aggrecan (Cat-315; MAB1581, MERCK; 1:1000), rabbit anti-aggrecan (AB1031, Millipore, Tokyo, Japan; 1:200), sheep anti-brevican (AF4009, R&D Systems; 1:200), goat anti-tenascin-R (AF3865, R&D Systems, Minneapolis, MN, USA; 1:200), mouse anti-PV (clone PARV-19, P3088; Sigma-Aldrich Japan, Tokyo, Japan; 1:1000), biotinylated Hyaluronic acid binding protein (HABP) (385911, MRECK, 1:200), mouse anti-HNK-1 (C6680, Sigma-Aldrich Japan, Tokyo, Japan; 1:200), and mouse anti-non-phosphorylated neurofilaments (SMI-32, Biolegend, San Diego CA, USA; 1:2000). The following secondary antibodies were used for visualisation: Alexa Fluor 488-conjugated goat anti-mouse immunoglobulin IgG (ab150113; Abcam, Cambridge, MA; 1:1000), FITC-conjugated anti-mouse IgM (sc-2082, Santa Cruz Biotechnology, Santa Cruz, CA, 1:1000), Texas Red-conjugated goat anti-rabbit (TI-1000; Vector Laboratories, Funakoshi Co., Tokyo, Japan), DyLight488-conjugated horse anti-goat IgG (DI-3088; Vector Laboratories; 1:500), Alexa Fluor 488-conjugated donkey anti-sheep IgG (A-11015; Thermo Fisher Scientific, Kanagawa, Japan; 1:1000), and streptavidin-conjugated Alexa Fluor 594 (S11227, Thermo Fisher Scientific; 1:1000).

### Microscopy imaging

Confocal laser scanning microscopy (LSM700; Carl Zeiss, Oberkochen, Germany) was used to quantify the density of Cat-315-, AB1031-, brevican-, tenascin-R-, WFA-, PV-, HABP-, and HNK-1-positive cells. Images (1024 × 1024 pixels) were saved as TIFF files using the ZEN software (Carl Zeiss) with a 10 × numerical aperture (NA = 0.45) or 20 × (NA = 0.8) objective lens and a pinhole setting corresponding to a focal plane thickness of less than 1 µm. Cat-315, AB1031, brevican, tenascin-R, WFA, PV, HABP, and HNK-1-positive cells were counted in a 1.0 × 1.2 mm area spanning all layers of the cortex. Before capturing, the exposure time, gain, and offset were carefully set to ensure a strong signal while avoiding saturation. Identical capture conditions were used for all the sections.

### Quantification of labelled PNNs

The brain regions were determined according to the mouse brain atlas of Paxinos and Franklin (2012). The data in the figures are presented according to the cortical layer profiles based on fluorescent Nissl staining (NeuroTrace 435/455 blue: N-21479; Molecular Probes, Thermo Fisher Scientific, U.S.). Microphotographs of the sections were captured using a 10 × or 20 × objective lens. All confocal images were saved as TIFF files and analysed using the NIH ImageJ software (Bethesda, MD; http://rsb.info.nih.gov/nih-image/). Twelve coronal sections containing the primary somatosensory cortices were selected from each mouse and stained. Only one area (region of interest) was analyzed in each of the 12 tissue sections from each mouse. Regions of interest that were predominantly barrel zones, rather than non-barrel zones, were selected for analysis. The stained neurones and PNN (neuronal soma size > 60 µm^2^) in the region of interest were manually tagged and counted. Background intensity was subtracted from the unstained portions of each section. The density of labelled neurons was calculated as cells/mm^2^. Quantification was performed by an independent observer blinded to the specimen's identity.

### Statistical analyses

The data are expressed as box plots with five animals per group. Statistical analyses were performed using the SPSS Statistics software (IBM Corp., Armonk, NY, USA). The data were tested for normal distribution using Bartlett's test. Data were analysed using one-way ANOVAs (for normally distributed data), and Kruskal–Wallis tests (for non-normally distributed data) were used to determine statistical significance. The data are presented as box plots. Statistical significance was defined as *p < 0.05 and ^+^*p* < 0.1.

## Results

### Development of PNNs in the somatosensory cortex of SAMP10 mice

**Fig. 1:**
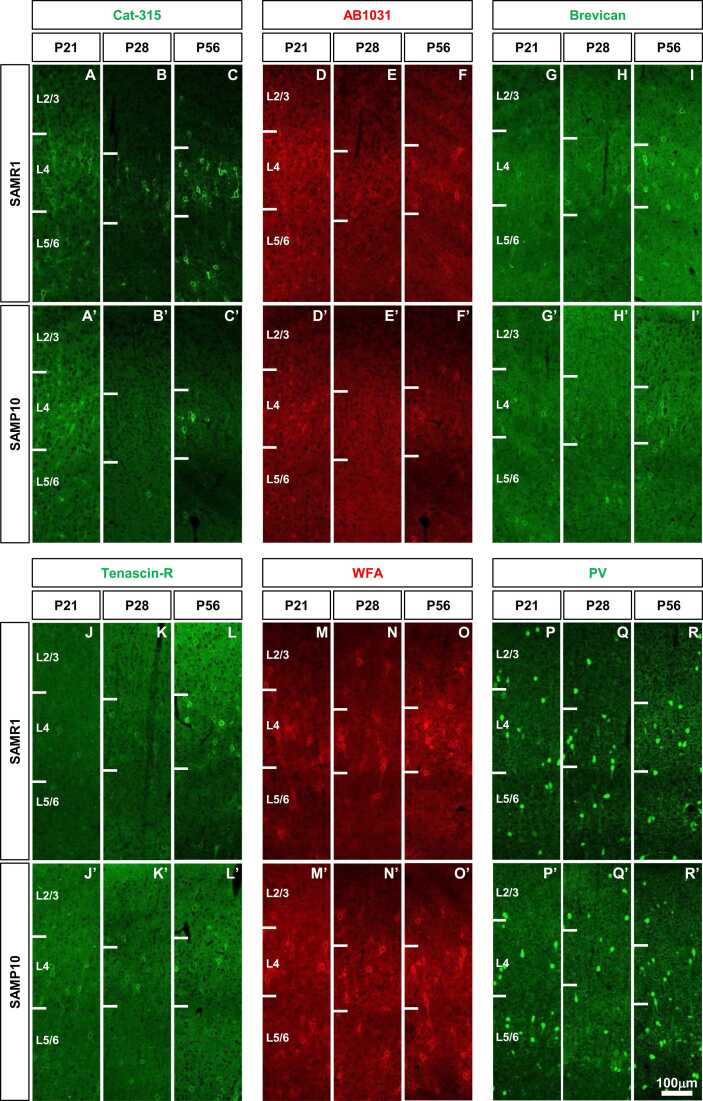
Developmental expression of PNNs in the primary somatosensory cortex of SAMP10 mice. Representative immunofluorescent images show the laminar distribution of Cat-315-positive PNNs (**A-C, A’-C’**), AB1031-positive PNNs (**D-F, D’-F’**), Brevican-positive PNNs (**G-I, G’-I’**), Tenascin-R-positive PNNs (**J-L, J’-L’**), WFA-positive PNNs (**M-O, M’-O’**), and PV-positive neurones (**P-R, P’-R’**) in the primary somatosensory cortex of SAMR1 (**A-R**), and SAMP10 (**A’-R’**) at P21, P28, and P56. Each image shows a coronal section cut through the primary somatosensory cortex (barrel zone) with the pia at the top. Scale bar = 100 μm in R’ (applies to **A–R**, **A’–R’**).

We first examined the expression of the PNN components in the postnatal brains of SAMP10 mice (Fig. 1). A few Cat-315-positive PNNs were observed in SAMR1 and SAMP10 mice at P21 (Fig. 1**A, A’**). Cat-315-positive PNNs at P21 had poor immunoreactivity. However, clear PNNs were observed at P28 (Fig. 1**B, B’**). At P28 and P56, a large number of Cat-315-positive PNNs were expressed in SAMR1 mice. However, it was expressed less in SAMP10 mice (Fig. 1**C, C’**). No obvious AB1031-positive PNNs were observed at P21 in either SAMR1 or SAMP10 mice (Fig. 1**D, D’**). At P56, AB1031-positive PNNs were observed in both SAMR1 and SAMP10 mice (**Fig. F, F’**). AB1031-positive PNNs expression was observed to be centered around L4 in both mice. At P21, Brevican-positive PNNs were observed as distinct PNNs in both SAMR1 and SAMP10 mice (Fig. 1**G, G’**). At P56, numerous Brevican-positive PNNs were observed in L2/3 and L5/6, mainly in L4, in both mice (Fig. 1**I, I′**). Tenascin-R-positive PNNs were observed as distinct PNNs in both SAMR1 and SAMP10 mice at P21 (Fig. 1**J, J’**). At P56, numerous distinct tenascin-R-positive PNNs were observed in L2/3 and L5/6, mainly in L4, in both mice (**Fig. L, L’**). Although WFA-positive PNNs at P21 were observed as indistinct PNNs in both mouse groups, they were plentiful (Fig. 1**M, M’**). At P28 and P56, numerous distinct WFA-positive PNNs were observed in L2/3 and L5/6, mainly in L4, in both mice (Fig. 1**N, O, N’, O’**). As PNNs form around PV-positive neurones, we also analysed PV-positive neurones in the somatosensory cortex. Numerous PV-positive neurons were observed in both mice at P21 (Fig. 1**P, P’**). Numerous PV-positive neurones were also observed in both mice at P56 (Fig. 1**R, R’**). When comparing the components of PNNs in the somatosensory cortices of SAMR1 and SAMP10 mice, clear differences were observed only in Cat-315-positive PNNs.

### Formation of PNNs in the somatosensory cortex of SAMP10 mice

**Fig. 2:**
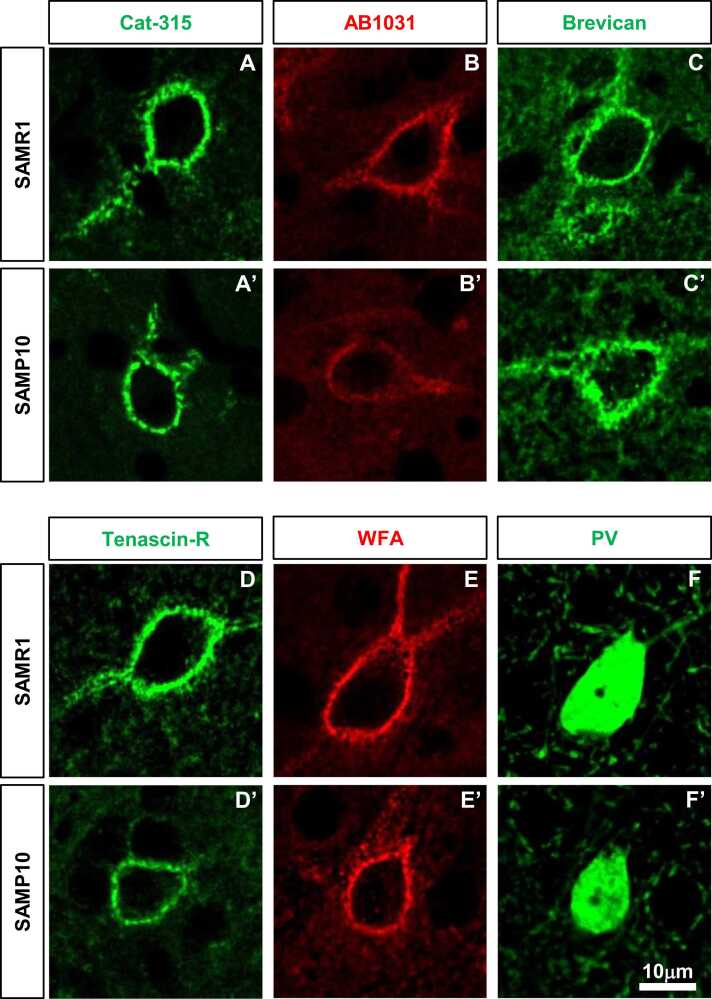
Morphological features of PNNs in the primary somatosensory cortex of SAMP10 mice on postnatal day 28. Representative immunofluorescent images show the characteristic mesh-like structure of Cat-315-positive PNNs (**A, A’**), AB1031-positive PNNs (**B, B’**), Brevican-positive PNNs (**C, C’**), Tenascin-R-positive PNNs (**D, D’**), WFA-positive PNNs (**E, E’**), and PV-positive neurons (**F, F’**) in layer 4 of the primary somatosensory cortex of SAMR1 (**A-F**), and SAMP10 (**A’-F’**) on postnatal day 28. Scale bar = 10 μm in F’ (applies to **A–F**, **A’–F’**).

We investigated whether there were any abnormalities in the formation and expression of PNNs in the somatosensory cortex of SAMP10 mice at the time of PNN formation (P28) (Fig. 2). Confocal analysis of individual PNNs revealed characteristic grid-like staining around the cell body, proximal portions of dendrites, and axonal origins. No clear difference was confirmed between SAMR1 mice and SAMP10 mice in Cat-315-positive PNNs (Fig. 2**A, A’**), AB1031-positive PNNs (Fig. 2**B, B’**), Brevican-positive PNNs (Fig. 2**C, C’**), tenascin-R-positive PNNs (Fig. 2**D, D’**), and WFA-positive PNNs (Fig. 2**E, E’**). As PNNs form around PV-positive neurones, we also analysed PV-positive neurones in the somatosensory cortex. No clear differences in PV-positive neurones were identified between SAMR1 and SAMP10 mice (Fig. 2**F, F’**).

### Quantitative changes in PNNs in the somatosensory cortex of SAMP10 mice

**Fig. 3:**
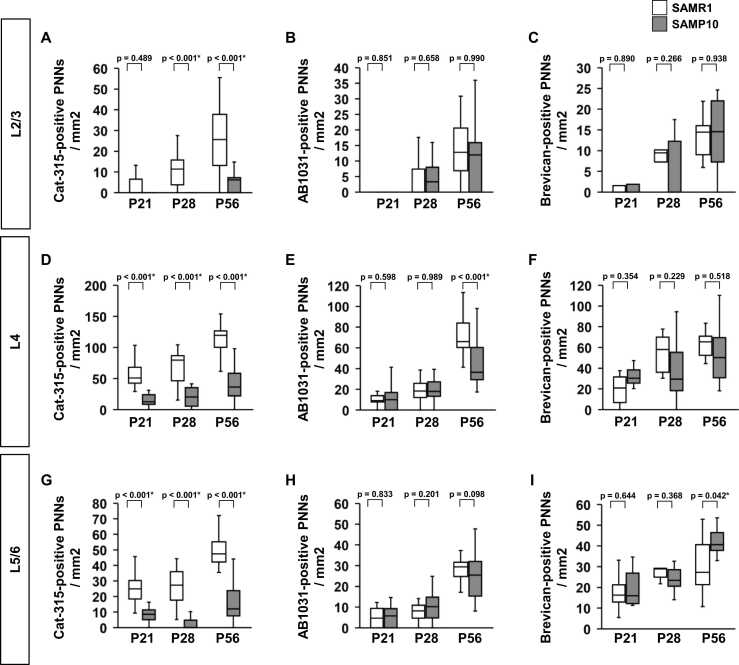
The density of Cat-315-positive PNNs, AB1031-positive PNNs, and Brevican-positive PNNs during postnatal development of SAMP10 mice. Layer-specific developmental patterns of Cat-315-positive PNNs density (**A, D, G**), AB1031-positive PNNs density (**B, E, H**), and Brevican-positive PNN density (**C, F, I**) in the individual layers of the primary somatosensory cortex. The data are presented as box plots (n = 5 mice per group). P-values were calculated using a one-way ANOVA. *p < 0.05, ^+^*p* < 0.1 for comparison of the same regions in SAMR1 and SAMP10 mice.

**Fig. 4:**
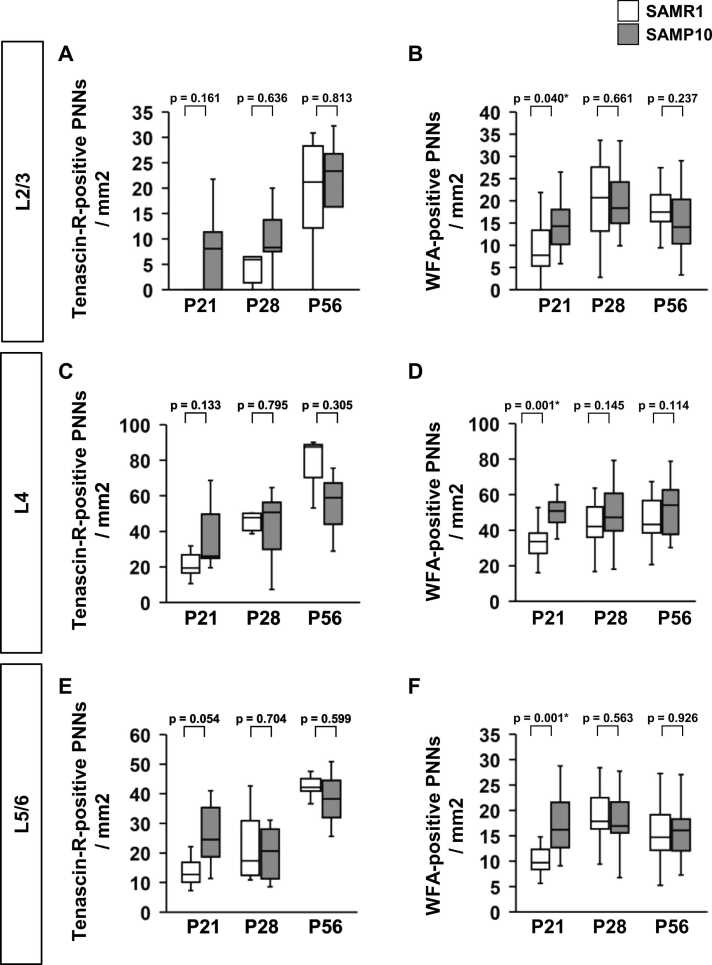
The density of Tenascin-R-positive PNNs, and WFA-positive PNNs during postnatal development of SAMP10. Layer-specific developmental patterns of tenascin-R-positive PNNs density (**A, C, E**) and WFA-positive PNNs density (**B, D, F**) in individual layers of the primary somatosensory cortex. The data are presented as box plots (n = 5 mice per group). P-values were calculated using a one-way ANOVA. *p < 0.05, ^+^*p* < 0.1 for comparison of the same regions in SAMR1 and SAMP10 mice.

The PNN numbers at P21, P28, and P56 were quantified separately in the subregions of the somatosensory cortex (Fig. 3, Fig. 4). At L2/3 of SAMP10 mice, the density of Cat-315-positive PNNs was lower at P28 and P56 than that of SAMR1 mice (Fig. 3**A**). At L4 and L5/6 of SAMP10 mice, the density of Cat-315-positive PNNs was lower at P21, P28, and P56 than that of SAMR1 mice (Fig. 3**D, G**). At P21, P28, and P56, there was no significant difference in the density of AB1031-positive PNNs at L2/3 and L5/6 between SAMP10 and SAMR1 mice (Fig. 3**B, H**). At the L4 of SAMP10 mice at P56, the density of AB1031-positive PNNs was lower than that of SAMR1 mice (Fig. 3**E**). At P21, P28, and P56, there was no significant difference in the density of Brevican-positive PNNs in L2/3 and L4 between SAMP10 and SAMR1 mice (Fig. 3**C, F**). At L4 of SAMP10 at P56, the density of Brevican-positive PNNs was higher than that of SAMR1 mice (Fig. 3**I**). There was no significant difference in the density of tenascin-R-positive PNNs in all the layers at P21, P28, and P56 between SAMP10 and SAMR1 mice (Fig. 4**A, C, and E**). In all layers of SAMP10 mice at P21, the density of WFA-positive PNNs was higher than that of SAMR1 mice (Fig. 4**B, D, and F**). There was no significant difference in the density of WFA-positive PNNs in all the layers at P21, P28, and P56 between SAMP10 and SAMR1 mice (Fig. 4**B, D, and F**).

### Morphology of PNNs in the primary somatosensory cortex of SAMP10 mice

**Fig. 5:**
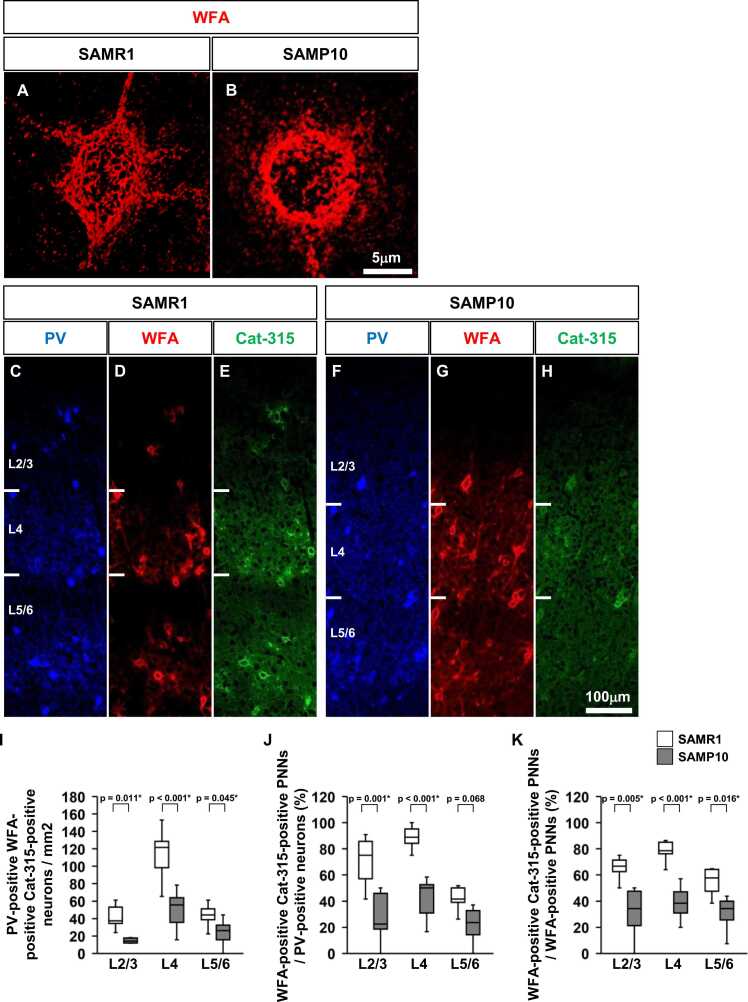
Morphological features of WFA-positive PNNs in the primary somatosensory cortex of SAMP10 mice on postnatal day 56. Confocal images of WFA-positive PNNs in layer 4 of the primary somatosensory cortex of SAMR1 (**A**) and SAMP10 mice (**B**) on postnatal day 56. Representative triple immunofluorescence images showing the distribution of PV-positive neurones (**C, F**), WFA-positive PNNs (**D, G**), and Cat-315-positive PNNs (**E, H**) in the primary somatosensory cortex (barrel zone) of SAMR1 (**C–E**) and SAMP10 mice (**F–H**). Region-specific patterns of PV-positive neurones co-localised with Cat-315-positive PNNs density (**I**), percentage of PV-positive neurones co-localised with WFA-positive- and Cat-315-positive PNNs (**J**), and percentage of WFA-positive and Cat-315-positive PNNs among all WFA-positive PNNs (**K**) in the primary somatosensory cortex are shown. The data are presented as box plots (n = 5 mice per group). P-values were calculated using a one-way ANOVA. *p < 0.05, ^+^*p* < 0.1 for comparison of the same regions in SAMR1 and SAMP10 mice. Scale bar = 5 µm in B (**A, B**), and 100 µm in H (**C–H**).

To investigate the structural features of PNNs in SAMR1 and SAMP10 mice, we completed three-dimensional reconstructions of WFA-positive PNNs in layer 4 of the primary somatosensory cortex at P56 (Fig. 5**A, B**). Three-dimensional reconstruction analysis of WFA-positive PNNs revealed a characteristic grid-like staining around the cell bodies and dendrites. However, no clear difference was observed in WFA-positive PNNs between SAMR1 and SAMP10 mice.

### Formation of Cat-315-positive PNNs and WFA-positive PNNs around PV-positive neurones in SAMP10 mice

To investigate the distributional characteristics of WFA-positive- and Cat-315-positive PNNs that formed around PV-positive neurones in SAMR1 and SAMP10 mice, we performed fluorescent triple staining at P56 (Fig. 5**C-H**). In SAMR1 and SAMP10 mice, PV-positive neurones, WFA-positive PNNs, and Cat-315-positive PNNs were mainly located in L4. Quantitative measurements were performed to confirm the co-localisation of PV-positive neurones, WFA-positive PNNs, and Cat-315-positive PNNs (Fig. 5**I-K**). In all layers of the somatosensory cortex of SAMP10 mice, the density of PV-positive neurones covered by WFA- and Cat-315-positive PNNs was lower than that of SAMR1 mice (Fig. 5**I**). In L2/3 and L4 of the primary somatosensory cortex of SAMP10 mice, the proportion of all PV-positive neurones covered by WFA- and Cat-315-positive PNNs was lower than that of SAMR1 mice (Fig. 5**J**). In all layers of the primary somatosensory cortex of SAMP10 mice, the percentage of WFA-positive and Cat-315-positive PNNs among all WFA-positive PNNs was lower than that of SAMR1 mice (Fig. 5**K**).

### HABP-positive PNNs in the primary somatosensory cortex of SAMP10 mice

**Fig. 6:**
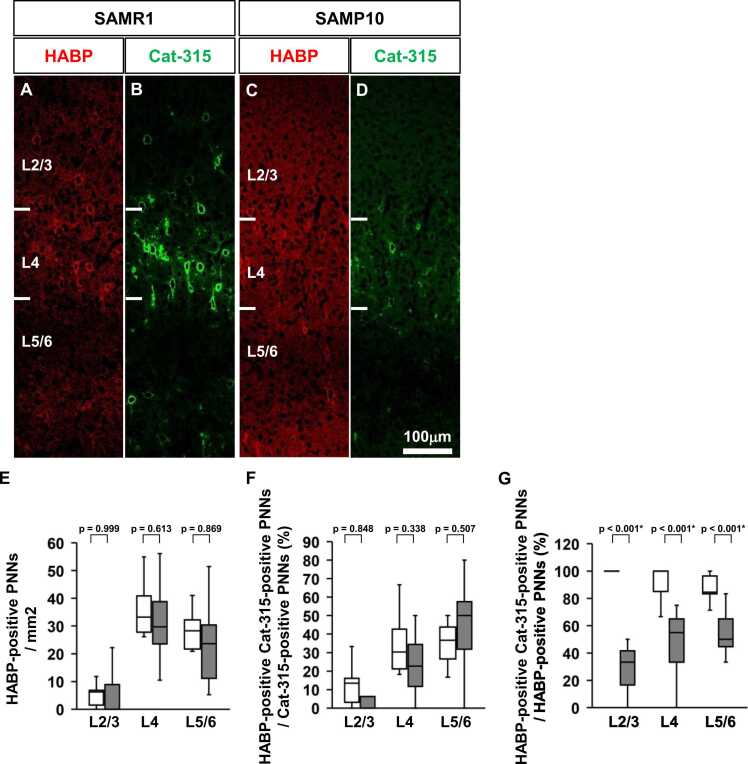
Distribution of HABP-positive PNNs and Cat-315-positive PNNs in the primary somatosensory cortex of SAMR1 and SAMP10 mice on postnatal day 56. Representative double immunofluorescence images show the distribution of HABP-positive PNNs (**A, C**) and Cat-315-positive PNNs (**B, D**) in the primary somatosensory cortex (barrel zone) of SAMR1 (**A, B**) and SAMP10 mice (**C, D**) on postnatal day 56. Quantitative analysis of HABP-positive PNNs in the primary somatosensory cortices of SAMR1 and SAMP10 mice. Region-specific patterns of HABP-positive neuronal density (**E**), percentage of HABP-positive and Cat-315-positive PNNs among all Cat-315-positive PNNs (**F**), and percentage of HABP-positive and Cat-315-positive PNNs among all HABP-positive PNNs (**G**) in the primary somatosensory cortex. The data are presented as box plots (n = 5 mice per group). P-values were calculated using a one-way ANOVA. *p < 0.05, ^+^*p* < 0.1 for comparison of the same regions in SAMR1 and SAMP10 mice. Scale bar = 100 µm in D (**A–D**).

Hyaluronic acid is one of the components of PNN. HABP binds hyaluronic acid, thus visualising PNN. We examined whether hyaluronic acid on the PNN of SAMP10 mice is different from that of SAMR1 mice. HABP-positive PNNs were mainly distributed in the L4 of the primary somatosensory cortex of SAMR1 and SAMP10 mice (Fig. 6**A-D**). Next, we performed a quantitative analysis of HABP-positive PNNs in the primary somatosensory cortex of P56 SAMP10 mice (Fig. 6**E-G**). No significant difference was observed in the density of HABP-positive PNNs between SAMP10 and SAMR1 mice in all layers of the primary somatosensory cortex at P56 (Fig. 6**E**). There was no significant difference between the two groups regarding the percentage of HABP-positive and Cat-315-positive PNNs among all Cat-315-positive PNNs in all layers of the primary somatosensory cortex (Fig. 6**F**). In all layers of the primary somatosensory cortex of SAMP10 mice, the percentage of HABP-positive and Cat-315-positive PNNs among all HABP-positive PNNs was significantly lower than that of SAMR1 mice (Fig. 6**G**).

### HNK-1-positive PNNs in the primary somatosensory cortex of SAMP10 mice

**Fig. 7:**
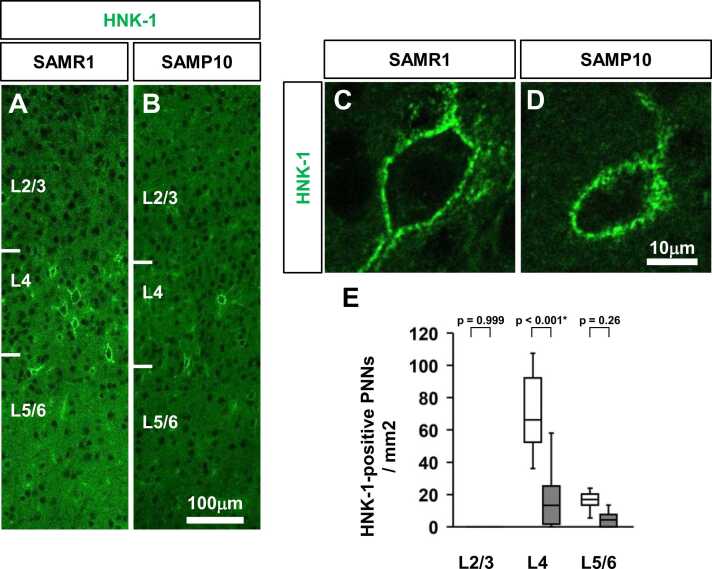
Distribution of HNK-1-positive PNNs in the primary somatosensory cortex of SAMP10 mice on postnatal day 56. Representative immunofluorescence images show the distribution of HNK-1-positive PNNs (**A, B**) in the primary somatosensory cortex (barrel zone) of SAMR1 (**A**) and SAMP10 mice (**B**) on postnatal day 56. High-magnification images of HNK-1 in the L4 of the primary somatosensory cortex of SAMR1 (**C**) and SAMP10 mice (**D**). Layer-specific patterns of HNK-1-positive PNN density (**E**) in the primary somatosensory cortex are shown. The data are presented as box plots (n = 5 mice per group). P-values were calculated using a one-way ANOVA. *p < 0.05, ^+^*p* < 0.1 for comparison of the same regions in SAMR1 and SAMP10 mice. Scale bar = 100 µm in B (**A, B**), and 40 µm in D (**C, D**).

Cat-315 recognises the human natural killer-1 (HNK-1) glycan epitope on aggrecan. Although HNK-1 epitopes have been reported to be highly expressed in the CNS (Voshol et al., 1996), there have been no reports on the use of anti-HNK-1 antibodies to detect HNK-1-expressing PNNs. Therefore, we visualised the HNK-1-expressing PNNs in the primary somatosensory cortex of SAMP10 mice using anti-HNK-1 antibodies (Fig. 7). We observed HNK-1-positive PNNs in L4 and L5/6 of the primary somatosensory cortex of SAMR1 mice (Fig. 7**A**). Although HNK-1-positive PNNs were present in L4 and L5/6 of SAMP10 mice, they were fewer than those of SAMR1 mice (Fig. 7**B**). HNK-1 positive PNNs in L4 of the primary somatosensory cortex were observed under high magnification (Fig. 7**C, D**). Confocal analysis of individual PNNs revealed characteristic grid-like staining around the cell body, proximal portions of dendrites, and axonal origins. No change was observed between SMR1 and SAMP10 mice regarding the existence of HNK-1-positive PNNs in mesh-like structures around the cell bodies. Next, we performed a quantitative analysis of HNK-1-positive PNNs in the primary somatosensory cortex of P56 SAMP10 and SAMR1 mice (Fig. 7**E**). At the L4 of the primary somatosensory cortex at P56, the density of HNK-1-positive PNNs was significantly lower in SAMP10 mice than in SAMR1 mice.

### Structure of PV-positive neurons in the primary somatosensory cortex of SAMP10 mice

**Fig. 8:**
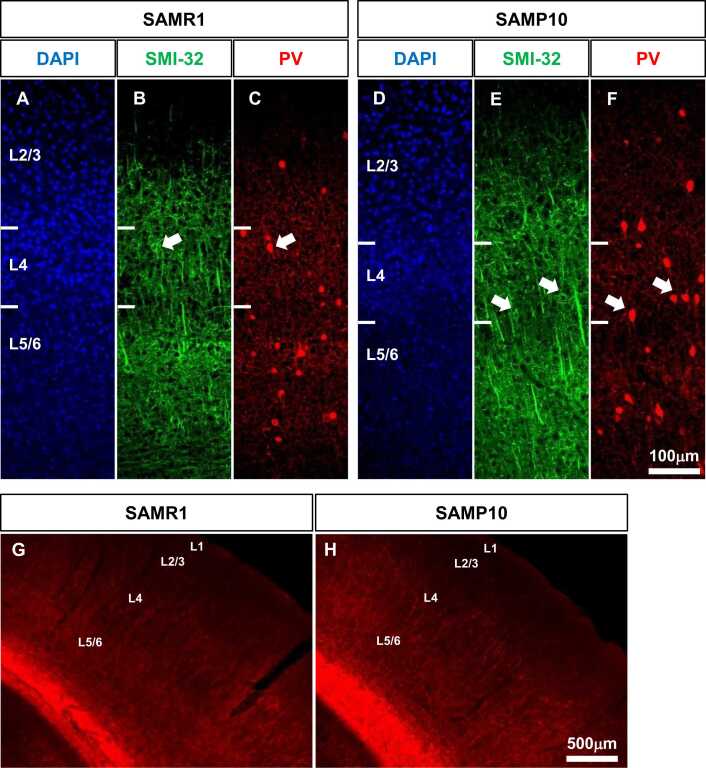
Distribution of non-phosphorylated neurofilament protein, and myelin basic protein in the primary somatosensory cortex of SAMP10 mice on postnatal day 56. Representative immunofluorescence images show the distribution of non-phosphorylated neurofilament proteins (**B, E**), DAPI (**A, D**), and PV-positive neurones (**C, F**) in the primary somatosensory cortex (barrel zone) of SAMR1 (**A–C**) and SAMP10 mice (**D–F**) on postnatal day 56. Arrows indicate SMI-32- and PV-positive neurons in layer 4. Representative immunofluorescence images show the distribution of myelin basic protein in the primary somatosensory cortex of SAMR1 (**G**) and SAMP10 mice (**H**). Scale bar = 100 µm in F (**A–F**), and 500 µm in H (**G, H**).

Cat-315-positive PNNs were formed around PV-positive neurones. We examined whether there was any difference in the structure of PV-positive neurones between SAMP10 and SAMR1 mice. L4 non-phosphorylated neurofilaments are present in PV-positive neurones (Del Río and DeFelipe, 1994, Van der Gucht et al., 2007, Horie et al., 2015). As the antibody SMI-32 recognises non-phosphorylated neurofilaments, we used SMI-32 to identify PV-positive neurones in the L4 of SAMP10 mice (Fig. 8**A-F**). No obvious abnormalities were observed in the expression of non-phosphorylated neurofilaments in the L4 between SAMP10 and SAMR1 mice.

It has been shown that the majority of myelin present in the neocortex and hippocampus primarily wraps around the axons of PV-positive neurons (Zonouzi et al., 2019). We visualised myelin using an anti-myelin Basic Protein (MBP) antibody to confirm its state in the primary somatosensory cortex (Fig. 8**G, H**). MBP expression was observed in L2/3, L4, and L5/6 of the primary somatosensory cortex, mainly in L4. By visual inspection, strong MBP expression was observed in L4, which is rich in PV-positive neurons, in both mice. MBP expression was then observed in L5/6, which has a high density of PV-positive neurons. No clear differences in MBP expression were observed between SAMP10 and SAMR1 mice by visual analysis.

## Discussion

In this study, we showed that the expression of Cat-315-positive PNNs in the primary somatosensory cortex of the SAMP10 strain was significantly lower than that of SAMR1. We also showed that there was no significant difference in many other components of the PNN between the SAMP10 and SAMR1 strains. This suggests that the SAMP10 strain has an abnormality limited to the Cat-315 epitope expression.

Cat-315 epitope expression was significantly decreased in SAMP10 mice from the early postnatal period up to adulthood. In previous reports, a significant decrease in the expression of the Cat-315 epitope was observed in the SAMP10 strain at 3 months of age (Saitoh et al., 2008). However, no expression of the Cat-315 epitope was observed at 12 months of age (Ueno et al., 2023). Blocking sensory input during early development results in decreased PNN formation in the sensory cortex (Sur et al., 1988, Guimaraes et al., 1990, Kind et al., 1995, Lander et al., 1997). However, it has no effect during adulthood (Sur et al., 1988). The formation of Cat-315-positive PNNs is said to depend on neuronal activity in both the barrel cortex and the superior olivary complex, as sensory deprivation markedly reduces the number of Cat-315-positive PNNs (Myers et al., 2012). Contrary to SAMR1 mice, SAM10 mice did not show any abnormalities in whiskers or vision. The other PNN components did not show a significant decrease in the SAMP10 line, contrary to those in the SAMR1 line. It is unlikely that somatosensory input was reduced in the SAMP10 strain. Therefore, SAMP10 mice indicate that the expression of Cat-315-positive PNNs is not dependent on sensory input. Further studies are needed to clarify which PNNs are dependent on sensory input.

Recent studies identified the sulfated glycosaminoglycan protein-binding region on aggrecan as a new type of HNK-1 epitope recognised by Cat315 (Yabuno et al., 2015). Sulfation of GlcA residues in the binding region prevents further elongation of the CS chains and reduces the number of CS chains on aggrecan in vitro. The HNK-1 epitopes are highly expressed in the CNS (Voshol et al., 1996). GlcAT-P knockout (PKO) mice have significantly reduced levels of HNK-1 epitopes, resulting in reduced long-term potentiation (LTP) in the hippocampal CA1 region and impaired spatial learning (Yamamoto et al., 2002). These phenotypes are likely due to impaired spinal maturation and cell-surface retention of glutamate receptors in the developing brain (Morita et al., 2009). HNK-1 carbohydrates synthesised by GlcAT-P are required for the proper functioning of the mammalian CNS.

In this study, HNK-1 expression was confirmed in the PNNs of SAMR1 mice; however, HNK-1 expression was significantly lower in SAMP10 than in SAMR1 mice. It has been shown that activity-dependent expression of bound HNK-1 epitopes may precisely control the number of CS chains on aggrecan, thereby modulating the functional properties of PNNs. The formation of Cat315-positive PNNs may be regulated by the expression of HNK-1 sulfotransferase (HNK-1ST), which transfers sulfate to the non-reducing terminal GlcA residue of the binding region (Senn et al., 2002, Morise et al., 2017). Forced expression of HNK-1ST increases the amount of bound HNK-1 epitope on aggrecan in vitro (Yabuno et al., 2015). ChGn-1 and HNK-1ST compete for the same substrate (a nonreducing terminal GlcA residue in the binding region). It has been reported that the balance between the expression and activity of these two enzymes is a possible mechanism for creating PNN heterogeneity in the mouse cortex. The decrease in Cat-315 and HNK-1 epitopes in the SAMP10 line may be the result of the decrease in HNK-1ST. Further studies are required to investigate the expression of HNK-1ST in the SAMP10 line.

The mouse SAMP10 strain developed by Shimada et al. exhibits age-dependent cerebral atrophy due to the age-dependent accumulation of DNA damage in cortical neurones and loss of synapses (Shimada et al., 1994, Shimada et al., 2002, Shimada et al., 2003). SAMP10 mice exhibit learning deficits accompanied by behavioural inhibition, which are thought to be caused by reactive oxygen species (ROS) (Miyamoto, 1997, Miyamoto et al., 1998). The relationship between learning deficits in SAMP10 mice and defects in the Cat-315 epitope of perineural aggrecan remains unknown. GlcAT-P knockout (PKO) mice with significantly reduced HNK-1 epitopes have reduced LTP in the hippocampal CA1 region and impaired spatial learning (Yamamoto et al., 2002). The learning deficits in the SAMP10 strain may also be due to HNK-1 epitope depletion. Further research is needed to investigate the relationship between the behavioural abnormalities reported to date in SAMP10 mice and the decrease in the Cat-315 epitope. HNK-1 is one of the glycans found on several neural cell adhesion molecules, transmembrane receptors, and extracellular matrix proteins in the nervous system, and has been shown to play a role in diverse neuronal functions, including cell recognition, adhesion, migration, synaptic plasticity, and preferential motor reinnervation and regeneration after peripheral and central nervous system injury (Morita et al., 2009, Castillo et al., 2021). Studies examining aging-related brain glycans using SAMP10 mice may contribute to the mechanisms of age-related disorders.

In the analysis in this study, no abnormalities were observed in the PNN components HABP, Brevican, and Tenascin-R in SAMP10 mice. Furthermore, no clear differences in expression were observed in non-phosphorylated neurofilaments present in PV-positive neurons in L4 of SAMP10 mice, and in myelin that mainly wraps around the axons of PV-positive neurons, compared to SAMR1 mice. It has been shown that the expression of PNN components is reduced in some neurodegenerative diseases (De Luca and Papa, 2016, Bitanihirwe et al., 2016). The expression of the Cat-315 epitope in PNNs in the SAMP10 mice used in this study was reduced, not absent. SAMP10 mice are useful for research into the function of the Cat-315 epitope in PNNs and its effect on PV-positive neurons.

This study focused on the somatosensory cortex. Somatosensory impairment can be either a long-term symptom or an acute symptom that lasts only a few days, depending on the underlying cause of the disorder. Symptoms include numbness, pain, and the inability to perceive the position of the limbs in space. Rehabilitation therapy has been shown to be an effective adjunct to pharmacological treatment for somatosensory impairment. Our findings suggest that rehabilitation therapy may help restore impaired neuronal activity by modulating the activity of pyramidal and PV-positive neurons (Tang et al., 2024). SAMP10 mice exhibit reduced Cat-315 epitopes around PV-positive neurons, which could lead to differential effects of rehabilitation therapy compared to controls. Somatosensory information is also essential for social behavior. Many children with autism spectrum disorder report hypersensitivity and/or hyposensitivity in multiple sensory domains (Crane et al., 2009). One theory regarding the cause of autism spectrum disorder is a reduction in the function of PV-positive neurons (Marin, 2012, Ferguson and Gao, 2018). This study may also contribute to understanding sensory dysfunction in autism spectrum disorders using SAMP10 mice.

The use of only male mice in our study may be considered a limitation. Further studies are needed to examine whether similar results can be obtained in female mice. In this study, only histological analysis was performed. Further research is needed to clarify the relationship between the behavioural and histological abnormalities observed in SAMP10 mice. In this study, the primary somatosensory cortex, where neuroplasticity has been widely studied, was the observational area, but further research is needed to see whether similar results can be observed in other brain areas. The SAMP10 mice exhibit age-dependent cerebral atrophy. Further studies are needed to investigate the relationship between the reduction in Cat-315 epitope observed in this study and the extracellular matrix and cerebral atrophy.

## Conclusion

In this study, we demonstrated that Cat-315 epitope expression is reduced during postnatal development in the primary somatosensory cortex of SAMP10 mice. No abnormalities were observed in the expression of other components of the PNN in SAMP10 mice compared to SAMR1 mice. Our findings indicate that SAMP10 mice have brain histological abnormalities from early life. Additionally, this will help us use SAM to elucidate the mechanisms of age-related abnormalities in brain function.

## List of abbreviations

ANOVA: analyses of variance

## Ethics approval and consent to participate

All animal experiments were performed in accordance with the U.S. National Institutes of Health (NIH) Guide for the Care and Use of Laboratory Animals (NIH Publication No. 80–23, revised in 1996) and approved by the Committee for Animal Experiments at the Kawasaki Medical School Advanced Research Centre.

## Declarations

none

## Authors’ contributions

All authors had full access to all study data and take full responsibility for the integrity of the data and the accuracy of the data analysis. Study concept and design: H. U., M. O., and T. I. Acquisition of data: H. U., Y. T., S. M., and E. K. Analysis and interpretation of data: H. U. and Y. T. Drafting of the manuscript: H. U. and M. O. Critical revision of the manuscript for important intellectual content: S. M., K. W., Y. T., and Y. M. Statistical analyses: H.U. and Y.T. Study supervision: M.O. and T.I.

## Funding

This study did not receive any specific grants from funding agencies in the public, commercial, or not-for-profit sectors.

## Consent for publication

Not applicable.

## CRediT authorship contribution statement

**Hiroshi Ueno:** Writing – original draft, Software, Formal analysis, Data curation, Conceptualization. **Yu Takahashi:** Software, Data curation. **Yosuke Matsumoto:** Supervision, Project administration. **Motoi Okamoto:** Writing – review & editing, Supervision, Conceptualization. **Takeshi Ishihara:** Writing – review & editing, Supervision, Conceptualization. **Sachiko Mori:** Data curation. **Eriko Kitano:** Data curation. **Shinji Murakami:** Supervision, Project administration. **Kenta Wani:** Supervision, Project administration.

## Declaration of Competing Interest

none

## Data Availability

The dataset is available on reasonable request from the corresponding author.
